# Innovations and Inequalities: The Challenge of Access in Multiple Myeloma Management in Brazil

**DOI:** 10.1016/j.htct.2026.107414

**Published:** 2026-09-24

**Authors:** Glaciano Nogueira Ribeiro, Edvan de Queiroz Crusoé, Angelo Maiolino, Vania de T. Moraes Hungria

**Affiliations:** a Clínica Hematológica - Oncoclínicas, Brazil; b Rede D’Or Oncologia, Salvador, Brazil; c Universidade Federal da Bahia, Brazil; d Universidade Federal do Rio de Janeiro, Brazil; e Instituto Américas de Pesquisa, Brazil; f Department of Hematology, Clínica São Germano, Brazil; g Brazilian Multiple Myeloma Group, Brazil (Grupo Brasileiro de Mieloma Múltiplo)

Multiple Myeloma (MM) is a hematologic neoplasm characterized by the clonal proliferation of plasma cells, which leads to monoclonal protein production, cytokine release, and clinical features such as anemia, renal impairment, and bone lesions. MM accounts for approximately 14% of all hematologic malignancies and 20% of deaths among hematologic cancers [1].

In Brazil, comprehensive official data regarding incidence, prevalence, and MM-related mortality remain limited. However, a published study involving 943 patients revealed a mortality rate 1.92 times higher in the public healthcare system than in the private sector [2]. To address this epidemiological data gap, the Brazilian Multiple Myeloma Group (GBRAM) has maintained a prospective registry since 2018, enrolling over 2,900 patients to date. More than 40 centers across both sectors contribute data, covering all Brazilian regions and spanning states home to approximately 90% of the national population, according to IBGE census data.

Data recently presented at the joint International Myeloma Society (IMS) and ABHH symposium reveal a mortality rate 2.14 times higher in the Brazilian public sector than in the private sector, supporting previous findings. Among transplant-eligible patients, this disparity is even more pronounced, with mortality 2.34 times higher in the Unified Health System (SUS). Patients in the public sector managed with intent to undergo first-line autologous hematopoietic cell transplantation achieved a median overall survival of 69.2 months. This outcome contrasts markedly with other real-world cohorts. In another Brazilian study, a comparable patient cohort achieved a median overall survival of 93 months in a combined public-private sample, whereas a Latin American cohort reported a median of 75.4 months. These figures reinforce that inequities in access and care infrastructure exert a profound impact on patient outcomes [[2], [3], [4]].

The past decade has seen remarkable progress in the availability of novel therapeutics for MM. Today, the private sector has access to monoclonal antibodies, bispecific antibodies, and Chimeric Antigen Receptor (CAR) T-cell therapy, alongside immunomodulators, proteasome inhibitors, corticosteroids, and conventional chemotherapy agents. These innovations have yielded marked improvements in efficacy, directly translating into extended overall survival.

In contrast, options for patients relying on public care through SUS remain largely restricted to conventional chemotherapy agents, corticosteroids, thalidomide, and bortezomib. Recently, the National Commission for the Incorporation of Technologies into the SUS (*Comissão Nacional de Incorporação de Tecnologias no SUS - Conitec*) approved a proposal submitted by the Brazilian Association of Hematology, Hemotherapy and Cellular Therapy (ABHH) to incorporate lenalidomide as maintenance therapy following autologous hematopoietic cell transplantation, an advance expected to yield substantial clinical benefit once fully implemented in clinical practice. Nevertheless, implementation barriers persist, as illustrated by carfilzomib; despite its approval for relapsed and refractory MM, a delay exceeding 800 days occurred between formal incorporation and real-world availability; it remains inaccessible across the vast majority of public centers.

Compounding the slow adoption of novel therapeutics are persistent structural deficiencies across the diagnostic landscape. Chronic barriers impede routine access to fundamental assays, including serum protein electrophoresis, immunofixation, and serum free light chain measurements. This delay in adopting diagnostic and monitoring technologies mirrors the lag in treatment availability, compromising accurate diagnosis, prognostic staging, and disease monitoring. Together, these systemic failures place public sector patients at nearly double the risk of mortality compared to those managed within the supplementary healthcare system.

Long considered an incurable disease, MM now offers tangible hope for long-term remissions, or even a functional cure, thanks to advanced cellular therapies, particularly CAR T-cell therapy. Recent long-term follow-up data demonstrate that one-third of patients treated with CAR T-cell therapy remain alive and progression-free five years post-treatment. Crucially, this durable response was achieved in a heavily pretreated population with a median of six prior lines of therapy, where 87% of patients were triple-class refractory and 42% were penta-refractory.

In conclusion, amidst rapid therapeutic innovation, foundational supportive care remains as vital as ever. Bisphosphonate therapy continues to be an essential pillar of comprehensive myeloma management. Likewise, maintaining up-to-date immunizations, implementing appropriate antimicrobial prophylaxis, and ensuring early identification and treatment of infectious complications are indispensable for safeguarding patient safety, preserving quality of life, and ultimately extending overall survival.

In summary, we are living in an era of unprecedented scientific optimism, where a potential cure for MM is becoming a tangible reality. However, the paramount challenge for Brazilian hematology transcends clinical trials, it is a critical issue of health equity. The concerted efforts of medical societies, researchers, and public health administrators must focus not only on generating robust evidence, but on accelerating real-world access to these technologies for all patients. Only when proven clinical efficacy is paired with equitable access, spanning from essential diagnostic assays to targeted and cellular therapies, can we eliminate the unacceptable mortality disparities currently dividing our healthcare system.

## Conflicts of interest

The authors declare no conflicts of interest.
